# High Precision Visual Dimension Measurement Method with Large Range Based on Multi-Prism and M-Array Coding

**DOI:** 10.3390/s22062081

**Published:** 2022-03-08

**Authors:** Xiao Zhou, Cong Zhou, Tingting Zhang, Xingang Mou, Jiaxin Xu, Yi He

**Affiliations:** 1 School of Mechanical and Electronic Engineering, Wuhan University of Technology, Wuhan 430070, China; zhouxiao@whut.edu.cn (X.Z.); c_zhou@whut.edu.cn (C.Z.); hahahuxian@whut.edu.cn (T.Z.); sunnymou@whut.edu.cn (X.M.); 3282383639@whut.edu.cn (J.X.); 2 Intelligent Transport Systems Research Center, Wuhan University of Technology, Wuhan 430063, China

**Keywords:** multiple prisms, M-array, high precision measurement, imaging system calibration, global coordinate coding

## Abstract

The visual dimension measurement method based on non-splicing single lens has the contradiction between accuracy and range of measurement, which cannot be considered simultaneously. In this paper, a multi-camera cooperative measurement method without mechanical motion is proposed for the dimension measurement of thin slice workpiece. After the calibration of the multi-camera imaging system is achieved through a simple and efficient scheme, the high-precision dimension measurement with a large field of view can be completed through a single exposure. First, the images of the edges of the workpiece are compressed and combined by splitting and merging light through the multi-prism system, and the results are distributed to multiple cameras by changing the light path. Then, the mapping relationship between the global measurement coordinates and the image coordinates of each camera is established based on the globally unique M-array coding, and the image distortion is corrected by the coding unit composed of black and white blocks. Finally, the edge is located accurately by edge point detection at the sub-pixel level and curve fitting. The results of measuring a test workpiece with the dimension of 24 mm × 12 mm × 2 mm through a single exposure show that the repeated measurement accuracy can reach 0.2 µm and the absolute accuracy can reach 0.5 µm. Compared with other methods, our method can achieve the large-field measurement through only one exposure and without the mechanical movement of cameras. The measurement precision is higher and the speed is faster.

## 1. Introduction

In modern production, there is a strong need for large field-of-view and high precision dimension measurement. The existing manual contact measurement method can meet the requirements of measurement range and precision at the same time, but the measurement efficiency is low and there are uncertainties caused by operators, environment, measuring equipment and other factors. To solve the problems of traditional contact manual measurement, some modern instruments such as coordinate measuring instruments, laser scanning measurements, optical microscope observation and measurement, using image processing technology to realize contour scanning, are improved and applied to the dimension measurement of the workpiece. These modern measuring instruments have obvious advantages, such as high precision and high stability, and their measurement accuracy can reach 1 µm or even 0.5 µm [1]. Using a coordinate measuring instrument (CMM) for contact measurement of the workpiece, the measurement accuracy is high, the stability is strong. However, the CMM is expensive, and being a contact measurement with measuring stress, the measurement process may cause destructive damage to the workpiece. Laser scanning measurement belongs to non-contact measurement, and the accuracy can reach the micron level. Generally, camera and motion mechanisms are needed for scanning measurement, and the time of high-precision measurement is very long [2,3]. The optical microscope has high measurement accuracy but low measurement efficiency, and professional detection personnel are required to complete the measurement task [4]. Some scholars have proposed the combination of a universal tool microscope and a charge-coupled device (CCD) to replace manual alignment reading [5]. However, due to the large magnification of the microscope, the all-dimensions measurement cannot be completed at one time. For the workpiece beyond the field of view, it needs to be measured several times, and the detection efficiency is still limited. Although these modern measurement technologies are simple in operation and high in digitization, the contradiction between measurement efficiency and cost still exists. At the same time, for large quantities of a workpiece, real-time automatic measurement cannot be completed and professional measuring personnel are required. Therefore, more efficient measurement methods need to be considered to achieve fast and high-precision measurement.

In recent years, microelectronics technology and image processing technology have developed rapidly [6], and scholars have conducted a lot of research in this field. Because vision-based measurement technology has the advantages of non-contact, high precision, large measurement range and online detection [7]. It has been more and more widely used in industrial geometric measurement, workpiece surface defect detection and workpiece surface deformation detection. The workpiece dimension measurement using machine vision technology, single-camera exposures to do measurement is suitable for low precision or the circumstances of a comparatively small measuring range. For the high-precision and large range measurement, multiple measurements need to be achieved by moving objects or cameras with mechanical movement devices [8], or multiple measurements need to be achieved by simultaneous exposure of different areas with multiple cameras, both of which require the splicing of multiple measurement results [9].

If a single camera is used for one-time imaging of the workpiece, the measurement field of view and resolution are closely related to the CCD device parameters. To keep the measurement precision unchanged and expand the measurement range, you can only use the algorithm of sub-pixel positioning [10,11]. The performance of the algorithm depends on the algorithm limit and the shape characteristics of the measured object. In the case of multiple imaging, precise position parameters between multiple fields of view need to be known, and external precise displacement measurement equipment or more complex calibration methods need to be used, which will increase the uncertainty of the system or reduce the measurement efficiency. In ref. [12], a planar array CCD and a high-precision measurement grating are used to calculate the actual position of the contour of the part, and a high-precision movement system is used to image the complete edge, realizing the precise measurement of the geometric quantity of the part, which can reach the detection level of 3 µm. In ref. [13], four cameras and continuous image data are used for matching to correct errors in measurement data to obtain the precision boundary of large-scale panels. In ref. [14], to get sub-pixel edge detection, a method based on convolution neural network (CNN) and Bi-directional long short-term memory is proposed, which overcomes the resolution limitation of image sensors and the influence of noise. In ref. [15], a completely constrained and model-free distortion correction method was proposed to solve the imaging distortion of a single-lens biprism system, which performed well in the distorted image data captured by the system.

In this paper, the prism assembly and M-array coding method are used to achieve the fast and high precision measurement of a large field of view by multi-camera collaboration. Firstly, a simple combination of prisms is used to change the light path, and the large area background irrelevant to the measurement results is abandoned. Only the regions that contribute to measurement are integrated into camera imaging by a designed optical path, which improves the utilization rate of the camera imaging plane and alleviates the contradiction between the field of view and resolution. The use of combined prisms disturbs the imaging light path of the whole field of view, which makes the imaging relationship of each camera complicated and brings some difficulties to system calibration. Next, the global coordinate system was established by using photo-etching glass board based on M-array, and the pixel-by-pixel mapping relationship was established by identifying the globally unique coding pattern in the M-array, and the camera distortion correction and parameter calibration were completed at one time. The prism assembly adopts some common triangular reflection prism and splitting prism, which is simple and easy to construct. High-precision and large-area photo-etching glass boards can be processed by mature photolithography technology, and the calibration of the entire complex measurement optical path system can be completed by single image analysis.

**Figure 1:**
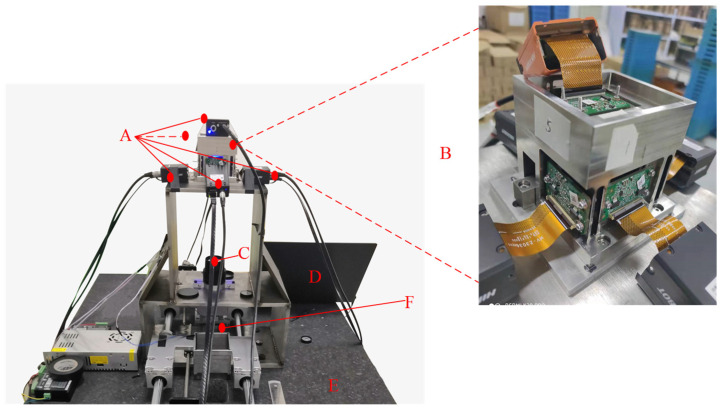
Actual picture of the test system. (**A**) Cameras; (**B**) prisms and cameras assembly; (**C**) camera lens; (**D**) computer; (**E**) marble slab; (**F**) photo-etching glass board and object to be measured.

The test system constructed by our method is shown in Figure 1. The marble slab is used as the base to reduce the influence of the environment and vibration on the prism imaging system. Fixed devices of the light source, prism system and lens are installed on a one-piece steel frame to accurately control the imaging focal length and object distance and increase the stability of the system.

The remainder of this paper is composed as follows: In Section 2, an enlarged imaging region method based on multi prism is proposed, and the design process and parameters of prism assembly are introduced in detail. In Section 3, the coordinate system transformation and calibration algorithm based on two-dimensional maximum area array (M-array) are introduced in detail. Section 4 is the experimental results and analysis of this paper. In Section 5, the feasibility and shortcomings of this method are discussed. Section 6 is a summary and conclusion of our work.

## 2. Multi-Prism Imaging Methods

To integrate the region of the workpiece which contributes to the measurement into the same camera to image, it is necessary to design a prism system to transform the optical path. It can improve the utilization rate of the camera imaging plane and solve the spatial interference between cameras. By splitting and merging the light of prisms, the prism system can compress and combine the parts of the workpiece which are out of field-of-view to make them imaged on the same camera without changing the imaging magnification. It alleviates the contradiction between accuracy and range of measurement.

### 2.1. Field of View Allocation

The high precision visual dimension measurement method proposed in this paper can be designed for high precision measurement of various shapes and various sizes of the workpiece while maintaining the measurement accuracy. Because this method carries out a single exposure to complete all the imaging required by the dimension calculation, and then performs multi-field stitching based on absolute coordinates. There is no need to move the camera or the target, which avoids the loss of accuracy and measurement time caused by moving. For the workpiece of different sizes and types, it is necessary to use multiple cameras to image each region of the target to be measured simultaneously, and each cameras’ field of view needs to be allocated for the target workpiece.

**Figure 2:**
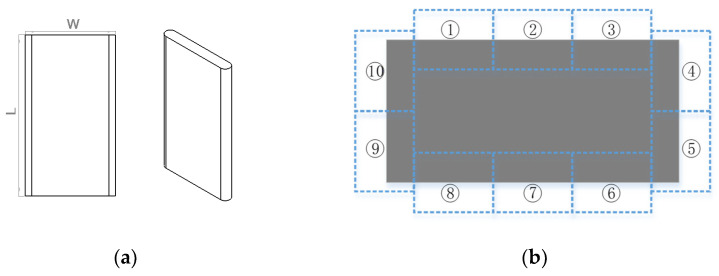
Oil pump blade and assignment of field of view. (**a**) Oil pump blade. (**b**) Field of view assignment diagram.

To facilitate the description of specific parameters in this method, a practical case is illustrated, in which the measurement object is an oil pump blade from an automobile. Its three-dimensional shape is shown in Figure 2a. The product length is about 24.940 mm, and the product width is about 12.962 mm, and its measurement precision requirements are ±1 µm. In this case, considering the requirements of measurement accuracy, there are few industrial cameras that can reach the measurement accuracy at the pixel level. The pixel size of existing industrial cameras is generally 1.4 μm ~ 14 μm, but the smaller the pixel size is, the smaller the sensor size will be under the same resolution, which is not conducive to placing the whole blade in the field of view. To avoid the measurement uncertainty caused by sub-pixel edge calculation as much as possible, the blade is designed to be magnified by 1.5 ~ 2× and the sensor size of the camera must be larger than 19.443 mm×37.41 mm ~ 25.924 mm×49.88 mm. To avoid the influence of diffraction phenomenon on the blade edge after magnified imaging, a blue parallel light source with a wavelength of 420 nm was selected.

According to the geometric dimension of the oil pump blade, it is only necessary to place each edge area of the blade in the camera field of view. The middle area of the blade that does not contribute to the measurement does not need to be imaged. According to the parameters of the existing industrial camera products on the market whose sensor size is generally 3.6 mm×4.8 mm ~ 13.5 mm×18.8 mm (1/3″ ~ 4/3″), at least 3 cameras are required to cover the edge areas in the length direction. To cover the edge areas while using the minimum number of cameras, three cameras with a sensor size of more than 1″ were selected in one length direction and two cameras with a sensor size of more than 1″ in one width direction. An MV-CH120-10UM Hikang camera was used. The camera is a grayscale array industrial camera with a sensor size of 1.1″, with a resolution of 4096 × 3000 and a single pixel size of 3.45 μm×3.45 μm. At least 10 cameras are needed to cover all four edges. The field of view allocation is shown in Figure 2b, where cameras need to be arranged from region ① to region ⑩ to complete simultaneous imaging.

### 2.2. Analysis of Multi-Prism Structure

The projected dimensions of a single industrial camera are generally 30 mm × 30 mm or more. Due to the small size of the workpiece relative to the external size of the camera, it is impossible to arrange the camera array closely without spatial interference for the field of view allocation diagram in Figure 2b. Therefore, it is necessary to expand the imaging light path in space, and a light splitting structure is designed to make multiple camera fields complete imaging at the same time. The optical spectroscopic structure in camera systems is mainly used in multi-spectral cameras and framing cameras to collect images of different spectra or different locations. The typical optical splitting is carried out by a splitting prism or splitting pyramid.

**Figure 3:**
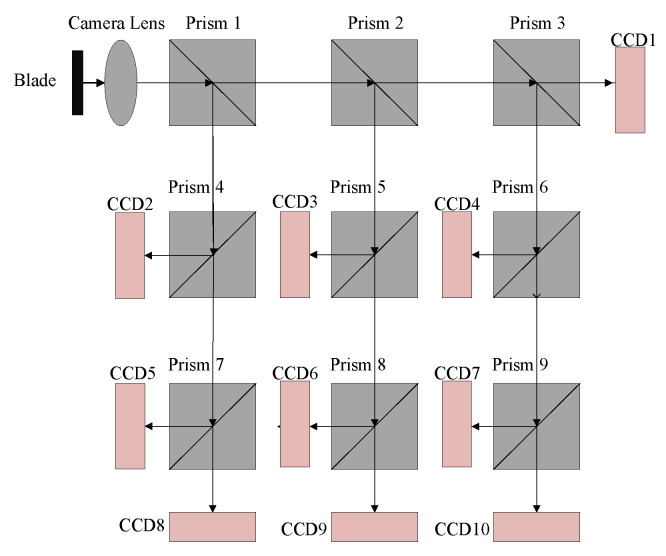
Prisms split the light.

In Figure 2b, the system is determined as a ten-amplitude system. Figure 3 is a schematic diagram of using multiple prisms to achieve ten frames, the size of the imaging plane in each camera field is 14.13 mm×10.35 mm. To allow different cameras to collect images in different fields, the center of the camera can deviate from the center of the prism. In this method, the space design should be compact and convenient for the assembly of various parts. As can be seen from Figure 3, when there are many fields to be segmented, the optical system will be very large and difficult to assemble.

**Figure 4:**
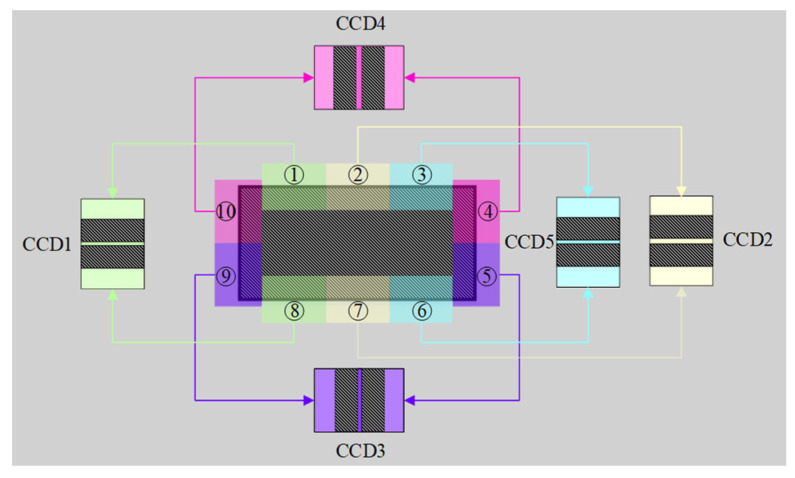
Prisms merge the light.

To reduce the number of cameras and improve the imaging plane utilization of a single camera, additional optical prism devices are designed. By using plane mirror combination, biprism or diffraction grating, the two opposite edge regions of the measured object can reach the same camera imaging plane through two different light paths, so as to achieve the purpose of merging light. As shown in Figure 4, area ① and area ⑧ are merged in the same camera imaging plane. With the similar combination of ②⑦, ③⑥, ④⑩, ⑤⑨, all edge imaging can be completed with five cameras. In a camera, the two opposite edges of the workpiece are collected simultaneously, and the two edges are distributed to the upper and lower parts of the camera photosensitive chip, respectively. This method will not reduce the measurement resolution of the system. On the contrary, because the middle part of the workpiece will not add an effective edge to the dimension measurement, the two opposite edge regions are presented in one camera to improve the measurement efficiency.

According to the existing research in [16,17,18,19] and the measurement object, the biprism structure or rhombic prism can be used to achieve multi-light path merging. It is difficult to adjust the angle between two prisms to achieve uniform distribution of camera image on the left and right and obtain clear image on both sides at the same time. Considering the limited space and convenient assembly, this paper uses the rhombic prism to complete light merging, which can avoid the angle adjustment and assembly problems in the prism system.

**Figure 5:**
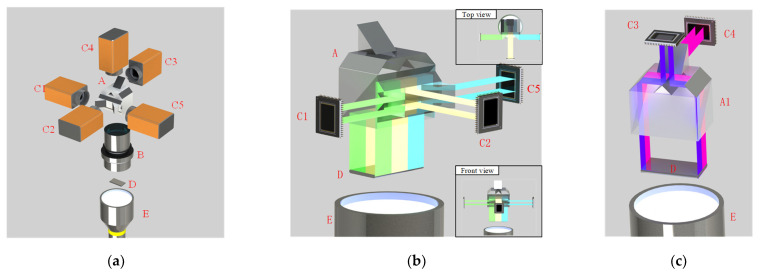
Schematic diagram of spatial distribution of imaging system and detail diagram of prism light path. (**a**) Spatial distribution of imaging system. (**b**) C1, C2 and C5 light path. (**c**) C3, C4 light path. (A) Prism assembly; (A1) part of prism assembly A; (B) camera lens; (C1–C5) cameras; (D) work piece to be measured; (E) parallel light source.

In this paper, in order to image the edge of the workpiece with complete coverage, five cameras and a prism system are used as shown in Figure 5a. The designed prism assembly A is located in the middle of the five cameras, which can split and merge light and ensure that the positions of the cameras do not interfere with each other. The blue parallel light source E is emitted from the bottom. After passing through the workpiece D to be measured, the light reaches the lens B of 55 mm caliber and becomes an enlarged image. After passing through the prism system A, the light is divided into two parts: one is deflected by 90°, as shown in Figure 5b, and the other is vertically upward, as shown in Figure 5c. Then, the light is merged to the camera imaging plane respectively. Camera C1, C2 and C5 image the long edge of the workpiece, while cameras C3 and C4 image the short edge of the workpiece. In Figure 5b,c, the lens is removed for rendering in order to show a clearer light path. In fact, the lens is always there for imaging.

In Figure 5b, green light is a section of the long side of the workpiece, which reaches the imaging plane of camera C1 through the prism assembly, yellow light is the light path to camera C2, and blue light is the light path to camera C5. To avoid the spatial interference of cameras C1, C2 and C5 placed side by side, two isosceles right-angle reflector prisms of 45° were used to change the direction of the light path received by cameras C1 and C5 and made them deflect by 90°. Therefore, cameras C2 and C5 are vertically distributed on both sides of camera C2. In Figure 5c, the purple light path is the light path to camera C3, and the pink part is the light path to camera C4. To show the details of cameras C3 and C4 better, we render only a part of Prism Group A. To avoid mutual spatial interference from cameras C3 and C4, one isosceles right-angle reflector prism of 45° is used to change the direction of the light path received by camera C4.

### 2.3. Calculation of Prism Parameters of Merged Light

**Figure 6:**
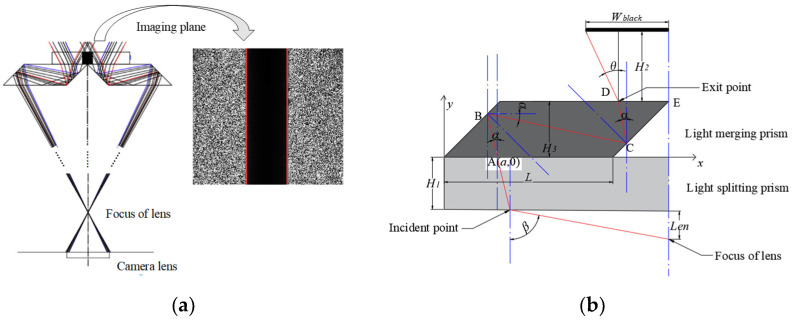
Rhombic prism model. (**a**) Rhombic prism imaging model. (**b**) Geometric parameter model.

The optical imaging model of the rhombic prism used for light merging is shown in Figure 6a. The light coming out of the camera lens into the prism are not parallel, so there is a black shadow region between the two red lines after the light is merged through a rhombic prism. After determining the spectral structure and spatial layout of multipath imaging, it is necessary to design the size of each prism in the imaging structure according to the actual dimension of the workpiece to image the effective edge of the workpiece, and discard the invalid background part. Firstly, the width of the dark shadow in Figure 6a needs to be calculated. It determines whether the two opposite edges of the workpiece can be captured in the same camera simultaneously and are not hidden in the dark shadow. Its calculation model is shown in Figure 6b.

The rhombic prism used for merging light is placed on the splitting prism. In Figure 6b, β represents the angle of incidence, Len represents the distance between the lens focus and the splitting prism, $H_{1}$ represents the height of the splitting prism, $H_{2}$ represents the distance between the upper surface of the rhombic prism and the imaging plane of the camera, $H_{3}$ represents the height of the rhombic prism, L represents the length of the rhombic prism, and $W_{black}$ represents half of the width of the black shadow. The light enters the splitting prism from the focal point of the lens and exits from the rhombic prism. The coordinate point A(a,0) represents the point at which light rays enter the rhombic prism. It can be calculated as:

(1) $$a=L+H_{3}-Len\times \left(\tan\beta \right)-H_{1}\times \left(\tan\alpha \right)\;$$

Black shadows are formed because light cannot be reflected from the vertex of the left boundary of the rhombic prism. Take the boundary position of the rhombic prism, when $a=H_{3}+H_{3}\times \tan\alpha$. After geometric calculations, we get the width of the black shadow:

(2) $$W_{black}=L\tan\alpha +H_{2}\times \tan(\arcsin(\lambda \sin\alpha ))$$

**Figure 7:**
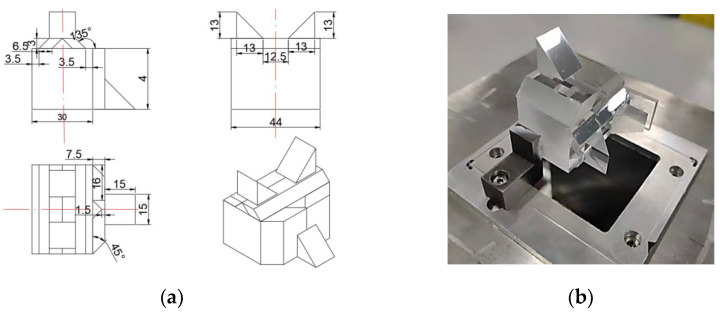
Detailed dimension picture and actual picture of prism assembly. (**a**) Dimension drawing of prism assembly; (**b**) Actual picture of prism assembly.

Finally, the prism assembly dimension was designed, as shown in Figure 7a below, and the actual picture was shown in Figure 7b. The height and length of the rhombic prism used for cameras C1, C2 and C5′s optical path combinations are 5 mm and 6.5 mm, the height and length of the rhombic prism used for cameras C3 and C4′s optical path combinations are 7.5 mm and 16 mm, and the height of the splitting prism is 30 mm.

## 3. Calibration Based on M-Array

The model of the prism imaging system is complicated because of the imaging distortion caused by manufacturing and assembly errors. To ensure the accuracy of measurement, point-to-point accurate correction is designed in this paper. In the system of prism splitting and merging light, a single camera can simultaneously image two regions outside the field of view, but the physical geometric distance of two regions in the same image is not known. To obtain the physical geometric distance between any pixel coordinates in the synthetic field of view, it is necessary to establish the corresponding relationship between the image coordinates and the actual physical coordinates.

**Figure 8:**
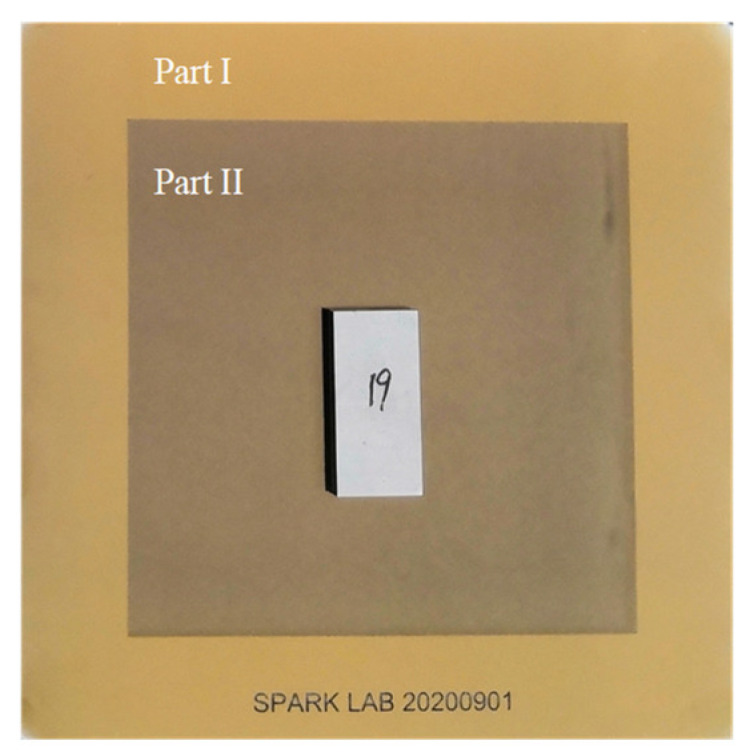
Actual picture of M-array photo-etching glass plate. Part I is the assembly area; part II is the M-array coding area, and the workpiece is placed in the coding area for the transformation of absolute coordinates.

Pseudo-random coding is a kind of random coding which can be determined in advance and implemented repeatedly. There are usually two forms of expression, a pseudo random sequence and a pseudo random array. The M-array is a typical pseudo-random array with specific window properties, often represented by U(u,v;m,n). In array of size (u,v), any sub-window of size (m,n) appears only once in the array [20]. Due to the global uniqueness of sub-windows, the sub-windows in an M-array can be used to encode the two-dimensional coordinate systems. The encoded M-array is printed on a 3-inch high-precision photo-etching glass plate, the minimum line width is 23 µm, as shown in Figure 8. The accuracy of the line width of the M-array photo-etching glass plate can reach 0.25 µm. By placing the measuring blade on the photo-etching glass board and imaging them at the same time, all the absolute physical coordinates of the blade can be obtained as well as all the edge pixel coordinates of the blade.

### 3.1. Calculate Corner Points Based on Minimum Classification Error

**Figure 9:**
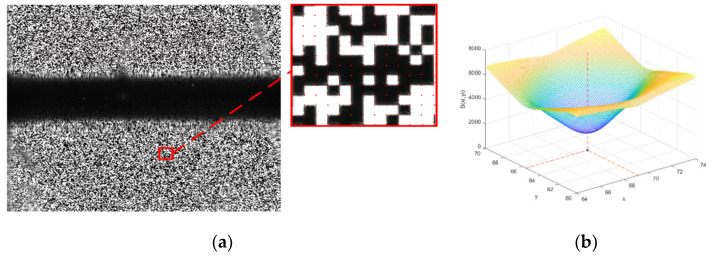
Image corner point extraction of photo-etching glass plate. (**a**) Corner point extraction.; (**b**) Corner point classification error distribution.

The local region of the M-array is shown in Figure 9a, we can see that the pattern encoded by M-array is not strictly alternating between black and white squares. There are continuous black blocks or continuous white blocks. So, the traditional pixel gradient descent method is not suitable for the corner point calculation of the system. To establish the M-array coordinate system and correct the image, this paper proposes the method of minimum classification error to calculate all the corner points under the M-array.

After the actual photo-etching glass board is imaged, the pixel size of a single black-or-white square is 10 × 10. To find the corner points between continuous white or black squares, it is necessary to use the squares in the surrounding area for auxiliary calculation. The minimum window of M-array designed in this paper is 5 × 5, which means that it is impossible for the M-array to have all black or white squares within 50 × 50 pixels. In this paper, we define parameter $S_{\left(x_{i},y_{i}\right)}$ as the square dispersion degree of pixel point $\left(x_{i},y_{i}\right)$. By taking $\left(x_{i},y_{i}\right)$ as the origin, divide the square at equal spacing within 50 × 50 pixels of its lower right corner, and then we can get the divided 25 squares and the total pixel value of each square. The dispersion degree of 25 pixels values is $S_{\left(x_{i},y_{i}\right)}\;$ at point $\left(x_{i},y_{i}\right)$.

The square dispersion degree in the 50 × 50 region is calculated for the possible locations of pixels, as shown in Equation (3). To obtain more accurate corner point coordinates, pixel interpolation within the range of $w_{A}\times h_{A}$ is carried out near point $\left(x_{i},y_{i}\right)$ in the image coordinate system, while f(x,y) is the pixel value, $\max Gate=w_{A}\times h_{A}\times 255,\;\min Gate=0$ and μ is the average pixel values in the region after interpolation. In the case of minimizing $S_{\left(x_{i},y_{i}\right)}$, the corresponding $\left(x_{\mathrm{dst}},y_{dst}\right)$ is the exact corner point required, where $N_{set}$ is the possible range of corner points, and $N_{set}$ is related to the initial value given, the maximum is 10 × 10 = 100. Figure 9b shows the distribution of $S_{\left(x_{i},y_{i}\right)}$ near the corner point $\left(x_{i},y_{i}\right)$, the blue point in the xoy coordinate plane is the final corner point position. The coordinates x and y corresponding to the minimum value of *z*-axis can be obtained to get the target corner point $\left(x_{\mathrm{dst}},y_{dst}\right)$.

$$\{\begin{array}{c} S_{\left(x_{i},y_{i}\right)}=\overset{x_{i}+w_{A}^{i}/2}{\underset{x=x_{i}-w_{A}^{i}/2}{\sum }}\overset{y_{i}+h_{A}^{i}/2}{\underset{y=y_{i\;}-h_{A}^{i}/2}{\sum }}s\left(x,y\right)\;\\ s\left(x,y\right)=\{\begin{array}{c} \max Gate-f\left(x,y\right)\begin{array}{cc} & f\left(x,y\right)>\mu \end{array}\\ f\left(x,y\right)-\mathrm{minGate}\;\begin{array}{cc} & others \end{array} \end{array} \end{array}$$

(3) $$\left(x_{dst},y_{dst}\right)=argmin\left|S_{\left(x_{i},y_{i}\right)}\right|\;i=0,1,\ldots N_{set}$$

The actual image of the photo-etching glass board and the corner points image obtained by searching are shown in Figure 9a, where the red point is the calculated sub-pixel corner point. There is no blade edge in the dark area, so there is no need to calculate the relevant corner points.

### 3.2. Coordinate System Transformation and Correction

**Figure 10:**
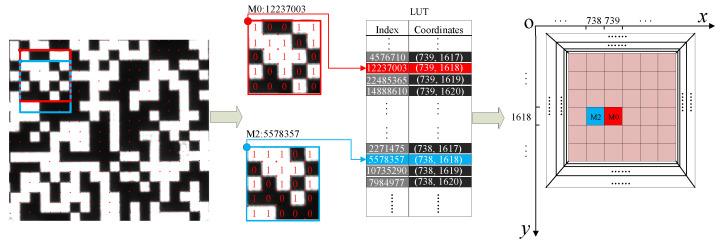
M-array decoding flow of camera C1.

The distortion model is no longer based on the traditional keyhole imaging model due to the existence of a prism system. The distortion parameters obtained by the traditional distortion model correspond to the global correction of the image. Obviously, the global distortion model described by finite parameters is not suitable for high-precision distortion correction occasions [21,22]. At the same time, the physical span of the imaging merging part is unknown due to the prism splitting and merging light, so the physical coordinates in the global range are obtained by using M-array decoding to calculate the span. The local M-array imaging decoding diagram is shown in Figure 10. Arbitrarily select two adjacent corner points calculated in Section 3.1. The coded coordinates of each corner point are made up of 5 × 5 squares in the lower right corner, with the white square representing 1 and the black representing 0. Due to the existence of prism and lens, the encoded image has the combination of mirror image and multiple rotations of 90°, so the decoding direction is different in different optical paths. For example, the 25-bit binary code in the optical path of camera C1, the lower right corner is the highest bit, the upper left corner is the lowest bit, and the numbers are arranged from top to bottom, and from right to left. For the red point, the decimal corresponding to the 25 bits binary is “12237003”. The physical coordinates obtained from the query table are (739, 1618), and the physical coordinates corresponding to the blue dot are (738, 1618). Through the pre-coded M-array coordinate system, the corresponding physical coordinates of all pixels in the imaging range can be obtained.

In this paper, it is necessary to establish the mapping relationship between the corner points and the physical coordinates of the M-array. Because the image coordinates and the physical coordinates of the M-array are in the same plane, the mapping relationship only includes rotation and translation. The transformation matrix is shown in Equation (4), where $\tilde{M}$ represents the coordinate system encoded by M-array, H represents the mapping matrix with a size of 3 × 3, $\tilde{P}$ represents the image coordinate system, and the introduced parameter s represents the transformation of any scale. The transformation matrix finally obtained satisfies Equation (5), which minimizes the transformation error. i represents the sequence number of extracted corner points, and Cnt represents the total number of corner points. According to the calculated transformation matrix, the corresponding physical coordinates of each pixel can be obtained. Moreover, the pixel coordinates of each corner point are corrected according to the physical coordinates encoded by the M-array.

$$\tilde{M}=\mathrm{sH}\tilde{P}$$

(4) $$\left[\begin{array}{c} x_{M}\\ y_{M}\\ 1 \end{array}\right]\begin{array}{cc} =s\cdot & \left[\begin{array}{ccc} h_{11} & h_{12} & h_{13}\\ h_{21} & h_{22} & h_{23}\\ h_{31} & h_{32} & 1 \end{array}\right] \end{array}\left[\begin{array}{c} x_{p}\\ y_{p}\\ 1 \end{array}\right]$$

(5) $$min\overset{Cnt}{\underset{i=0}{\sum }}{\left(x_{M}^{i}-s\frac{h_{11}x_{p}^{i}+h_{12}y_{p}^{i}+h_{13}}{h_{31}x_{p}^{i}+h_{32}y_{p}^{i}+1}\right)}^{2}+{\left(y_{M}^{i}-s\frac{h_{21}x_{p}^{i}+h_{22}y_{p}^{i}+h_{23}}{h_{31}x_{p}^{i}+h_{32}y_{p}^{i}+1}\right)}^{2}$$

To avoid the gross error caused by the calibration of a single frame, the optimal transformation matrix was calculated by synthesizing the mapping parameters of multiple M-array images taken at different positions. We put the M-array board in the same plane, rotate and translate arbitrarily, and take N clear pictures. The spatial position relationship is described by the affine transformation model, as shown in Equation (6). $P_{i}$ represents the corrected corner points pixel coordinates of the i-th image, and $A_{i}$ represents the affine transformation coefficient matrix from the i-th frame corner point coordinate to the first frame corner point coordinate.

$$A_{i}\cdot P_{i}=P_{1}$$

(6) $$\left[\begin{array}{ccc} a_{i} & b_{i} & c_{i}\\ d_{i} & e_{i} & f_{i}\\ 0 & 0 & 1 \end{array}\right]\left[\begin{array}{c} x_{i}\\ y_{i}\\ 1 \end{array}\right]=\left[\begin{array}{c} x_{1}\\ y_{1}\\ 1 \end{array}\right]$$

To get optimal affine parameters, the corresponding parameters under the minimum error should be calculated as Equation (7). The N−1  frames of the image are sequentially affine transformed to the first frame, and N−1 affine matrices A are obtained. In Equation (8), to eliminate the gross error, the obtained pixel matrix is averaged. $P^{*}$ represents the image coordinates after N frame adjustment. The mapping matrix H from pixel coordinate system to physical coordinate system can be recalculated according to $P^{*}$ instead of $P_{1}$ in Equation (4).

(7) $$A_{i}=argmin||A_{i}\cdot P_{i}-P_{1}|{|}^{2}$$

(8) $$P^{*}=\frac{\sum_{i=1}^{N}A_{i}\cdot P_{i}}{N}$$

**Figure 11:**
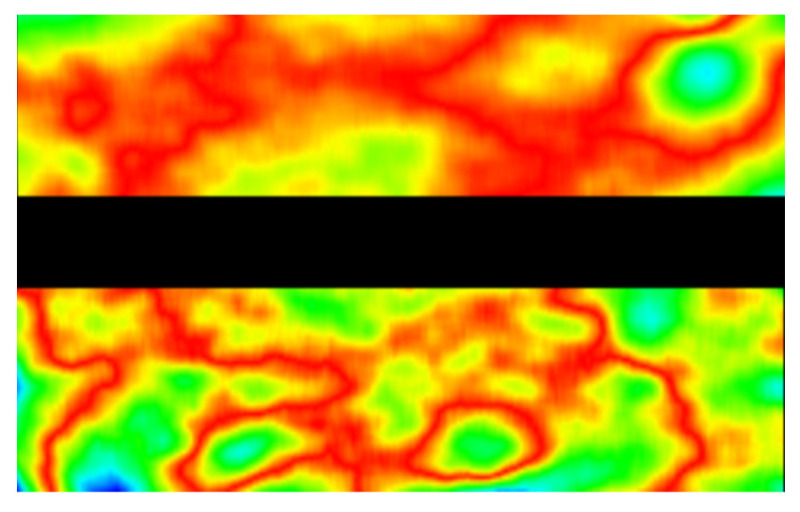
Distortion distribution. Red represents high distortion, followed by green and blue.

After the corrected corner point is obtained, the distance between the corrected corner and the original corner is calculated, and the distorted thermal map is drawn as shown in Figure 11. Due to the light merging effect of the rhombic prism, there are black shadows in the center of the image, so distortion statistics are not included. The red part represents the largest distance gap, followed by blue and green. As can be seen from Figure 11, the distortion is small near the image center, then becomes larger with the increase of the distance from the center. However, due to the existence of manufacturing and assembly errors of the prisms, the distortion presents discrete irregularity. In view of the actual image distortion, it is difficult to accurately describe it by the traditional global formula. For high precision measurement, it is necessary to map point by point to get an accurate coordinate system transformation relation.

## 4. Experimental Results and Analysis

**Figure 12:**
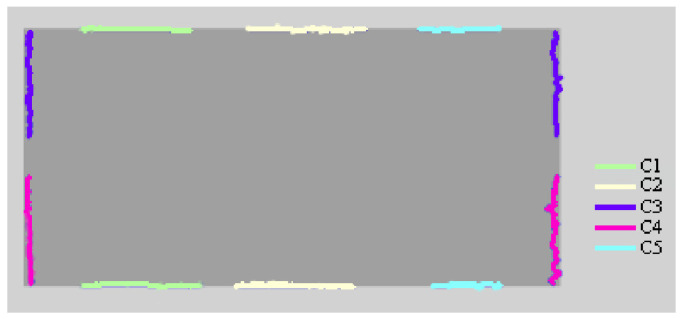
All the edge points are converted to the M-array coordinate system.

The workpiece to be measured is placed in the measurement area, and the edges of the five fields of view are converted to the M-array coordinate system and drawn into the same image, as shown in Figure 12. In order to visualize the edge details, adaptive amplification is performed in the *Y*-axis direction for the edge parallel to the *X*-axis and in the *X*-axis direction for the edge parallel to the *Y*-axis.

Since the accuracy of M-array calibration directly affects the final result of the whole measurement system, this paper verifies the accuracy of the calibration method. Because the object to be measured and the M-array are always in the same plane, the dimension calculation of the object to be measured depends on the corner extraction of the M-array and the accuracy of the coordinate system transformation. The accumulated manufacturing error of the photo-etching glass board that can cover the field of five cameras is within 0.25 µm, so we believe that all the corner points in the same row or column lie on a physical straight line. Due to the existence of errors from the corner point extraction algorithm and errors caused by distortion, extracted corner points deviate from the linear discrete distribution.

**Figure 13:**
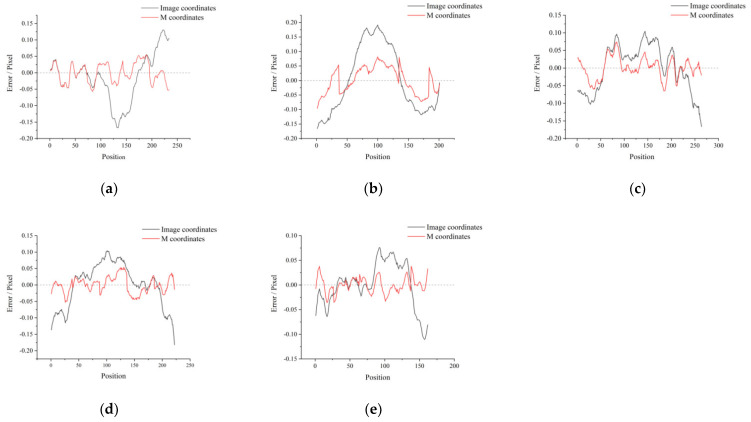
Distribution of corner points on a straight line in five camera images respectively. (**a**–**e**) corresponds to cameras C1–C5 respectively.

For the M-array images captured by five cameras, a random row of corner points is extracted, respectively, and the distribution in the image coordinate system is shown as the black line in Figure 13. Through the coordinate system transformation established by our method, it corresponds to the M-array coordinate system, as shown in the red line in Figure 13. Figure 13a–e correspond to cameras C1–C5 respectively. Due to the existence of corner point extraction and distortion error, the corner point distribution fluctuates in the range of straight line ±0.2 pixels. It is mapped to the decoded physical coordinate system to remove the distortion, and then converted to the same ordinate pixel unit. The corner points fluctuate in the range of ±0.1 pixels, that is ±0.2 µm. Since the corner point is calculated based on minimum classification error, so there are still subtle fluctuations in the M-array coordinate system.

**Figure 14:**
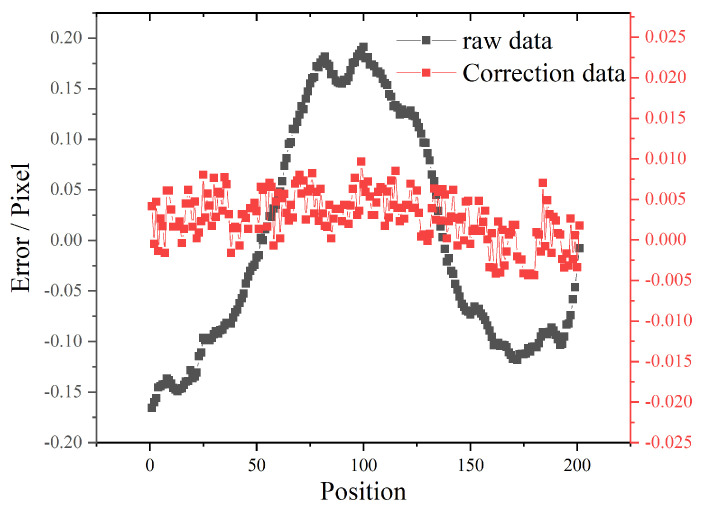
Corner points correction of image coordinate system.

Assuming that the error of corner point calculation conforms to normal distribution, the least square fitting is carried out to remove the error caused by corner point calculation in the physical coordinate system, so a standard straight line in the physical coordinate system is obtained. Then, the straight line of this physical coordinate is inversely corresponding to the pixel coordinate. For camera C2, the corrected pixel corner coordinate curve is shown as the red line in Figure 14. It can be seen that the red line completely converges within the range of ±0.01 pixel, which indicates that the coordinate system transformation relationship is accurate and corresponding. For the other four cameras, we also conducted the same corner points data tests, and the transformation relationship between pixel coordinates and physical coordinates could meet the accuracy requirements completely.

**Figure 15:**
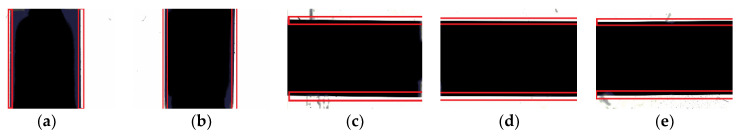
Simultaneous imaging of five cameras. (**c**–**e**) were the pictures imaged by cameras 1, 2 and 5; (**a**,**b**) were the pictures imaged by cameras 3 and 4.

Then, we used the established table of the corresponding relationship between pixel coordinates and the physical coordinate system to measure blades. The blade was simultaneously imaged in five cameras as shown in Figure 15. The length is calculated from Figure 15c–e imaged by cameras 1, 2 and 5, and the width is calculated from Figure 15a,b imaged by cameras 3 and 4. The red rectangular box is the blade edge region used in the calculation.

**Figure 16:**
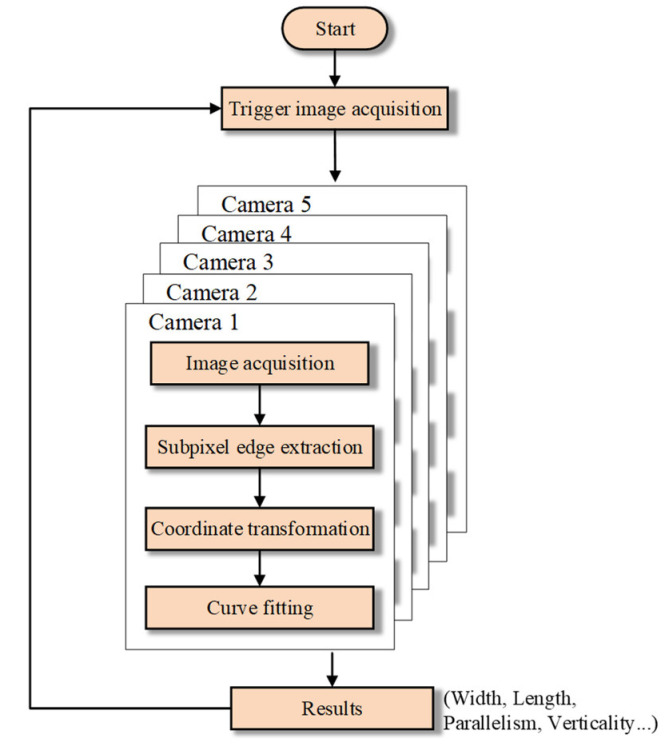
Algorithm flow of dimension measurement.

The main algorithm flow chart of the measurement part is shown in Figure 16. Due to the high measurement precision, it is necessary to give trigger signals to five cameras at the same time to avoid the influence of the external environment, so that each camera starts imaging at the same time. Then, sub-pixel edge extraction and coordinate system conversion are carried out for five images respectively. Finally, all the blade edge points converted to the M-array coordinate system are fitted and the final results are calculated and output.

Two experiments were carried out to evaluate the accuracy and precision of the proposed method: one workpiece was measured repeatedly to compare the results; 100 workpieces were measured and compared to the reference value obtained by a professional contact measuring instrument.

**Figure 17:**
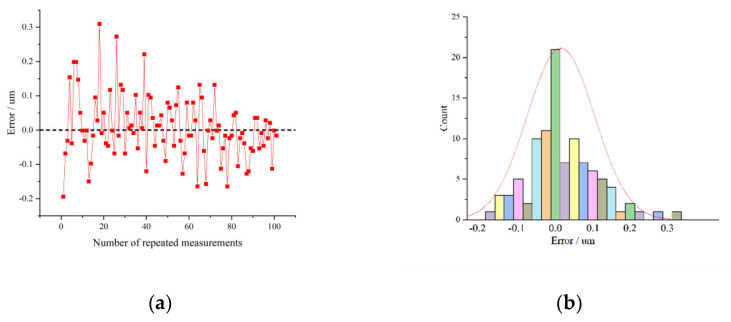
Errors of repeated measurement. (**a**) Repetition errors distribution; (**b**) Repetition errors histogram.

First, the same blade is placed in different positions in the field of vision and measured 100 times repeatedly. The consistency error distribution is shown in Figure 17a and the error probability distribution is shown in Figure 17b. 97% of the measurement results fluctuate within 0.2 µm. The variation could be from random changes of the external environment, including temperature, humidity, stability of light source, etc., as well as errors caused by the coordinate conversion of different positions.

**Figure 18:**
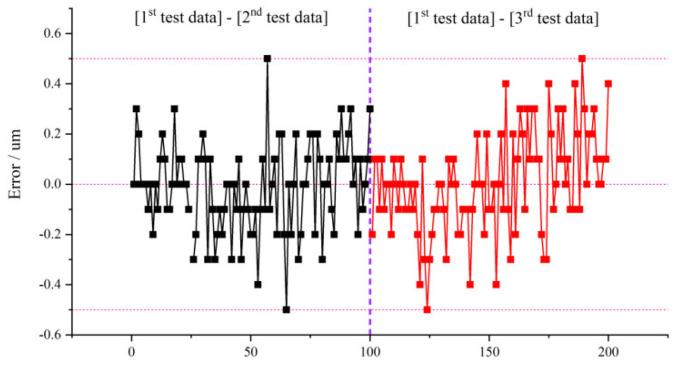
Repeated measurement error of different blades.

Then, the measurement of the system was carried out three times for 100 different blades, respectively, at different times. Multiple measurements were conducted by professional surveyors and instrument on 100 blades, and the average measurement results are used as the reference data. The repetitive measurement errors of the 100 blades were obtained by subtracting the second and third test data, respectively, from the first one, as shown in Figure 18. The repeated measurement error distribution of 100 different blades is within 0.5 µm, indicating that the batch of measured blades has little impact on the stability of the system.

**Figure 19:**
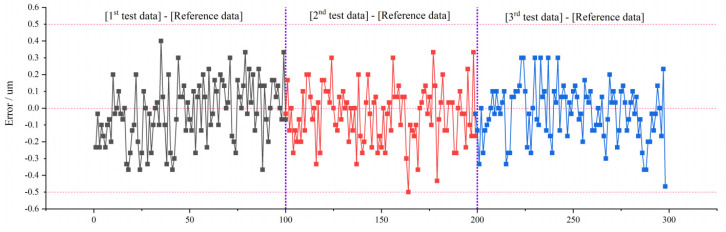
Measurement errors of blades.

The three measured values were compared with the reference values, respectively, and the error distribution was shown in Figure 19. In Figure 19, abscissa 0–100 is the difference between the first test data and the reference data, 100–200 is the difference between the second test data and the reference data, and 200–300 is the difference between the third test data and the reference data. The ordinate unit is µm, and it can be seen that errors between the measurement results of our method and the reference values is within 0.5 µm, which can meet the accuracy requirements.

There are several methods of high precision dimension measurement based on vision, and the comparison of several methods is shown in Table 1. Among them, the high magnification of the universal tool microscope can achieve the plane measurement accuracy up to 0.31 µm, but its field of view limits its range to 0.76 mm only. The high resolution of the grating scale has an absolute advantage in two-dimensional displacement measurement. When used for dimensional measurement, the accuracy can reach 0.7 µm, the measurement range is up to 1.1 mm. Using the principle of laser triangulation for visual measurement, accuracy can reach 10 µm, measurement range can reach 250 mm. The blade workpiece can be measured manually with a twist spring instrument. The error is 1um, but it has an artificial uncertainty error. The measurement accuracy of the method in this paper can reach 0.5 µm for 25 mm, and due to the universality of the method, the measurement accuracy can also be guaranteed for a larger range of dimension measurement in principle. For the test system used in this paper, the maximum measurement field of view can reach 30.4 mm×22.8 mm. From the comprehensive comparison of precision and range of measurement, the proposed method has higher advantages than the existing high precision plane geometry measurement scheme.

## 5. Discussion

This paper proposes a visual dimension measurement method based on multi-prism splitting and merging light which can achieve the multi-camera cooperative measurement through a simple and efficient scheme without mechanical movement. For cuboid blades in the above case, the 100 times repeatable measurement uncertainty of the same blade is within 0.2 µm. For the comparison between the measurement results of our method of 100 different blades and the reference values, the measurement uncertainty is within 0.5 µm, which can meet the precision requirements. The feasibility of this method for large measurement and high precision workpiece measurement is verified through multiple tests on different test samples. For the measurement of a thin slice workpiece without mechanical movement, a single exposure can realize the measurement of a large field of view, and the measurement precision is higher and the speed is faster than the traditional scheme. Due to the rapid non-contact detection of visual measurement, this method can also be equipped with corresponding mechanical picking, transporting and sorting devices, which can carry out automatic measurement work with high efficiency and high precision in large quantities.

As the measurement accuracy is below 1um, stability between imaging structures is required. The slight displacement of mechanical structure caused by ambient temperature, humidity and airflow may bring errors to the final results. Our follow-up work should be to continuously test and collect data to compensate the measurement results for environmental factors. For the measurement object with a large thickness, in order to ensure the measurement accuracy, we also need to expand the depth of field and reconstruct the image at different depths. We will conduct further research and exploration on the method of a measured object whose thickness exceeds the measured depth of field.

## 6. Conclusions

For the contradiction between accuracy and range of traditional visual methods, we discard the large region background irrelevant to the measurement results to maximize the use of the imaging plane of cameras. The edge image that contributes to measurement can be compressed and merged by splitting and merging the light of multiple prisms. Then, the global coordinate coding board based on M-array is constructed by using the unique global characteristics of M-array. The coded board and the workpiece are placed on the same plane to establish the mapping relationship between the global measurement coordinates and the image coordinates of each camera. Because the continuous M-array coordinate system can cover the whole field of view of the workpiece measurement, the high precision correction of camera distortion and parameter calibration can be completed at one time. Finally, the edge position and dimension measurement results are obtained by detecting sub-pixel edge points and curve fitting. Compared with other traditional visual measurements, our method is more versatile for large-scale and high-precision measurements. For different types of measurement objects, only the corresponding prism combination needs to be designed, and other parts do not need any change.

## Figures and Tables

**Table 1:** Method in this paper is compared with other methods.

Method	Accuracy/Measuring Range
Universal tool microscope combined with CCD [23]	0.31 µm/0.76 mm
Raster coded measurement [24]	0.7 µm/1.1 mm
Machine vision Measurement based on laser triangulation [25]	10 µm/250 mm
Manual twist spring instrument measurement	1 µm/25 mm
Method in this paper	0.5 µm/30.4 mm

## Data Availability

Data collected through research presented in the paper are available on request from the corresponding author.
